# Rodent Models Assessing Mammary Tumor Prevention by Soy or Soy Isoflavones

**DOI:** 10.3390/genes10080566

**Published:** 2019-07-26

**Authors:** Roger A. Moorehead

**Affiliations:** Department of Biomedical Sciences, Ontario Veterinary College, University of Guelph, Guelph, ON N1G2W1, Canada; rmoorehe@uoguelph.ca

**Keywords:** soy, isoflavones, mammary tumor prevention, rodent models, chemical carcinogens, transgenic mice

## Abstract

While epidemiological studies performed in Asian countries generally show that high levels of dietary soy are associated with reduced breast cancer risk, studies in Western countries have typically failed to show this correlation. In an attempt to model the preventative actions of soy on mammary tumor development, rodent models have been employed. Thirty-four studies were identified that evaluated the impact of soy products or purified soy isoflavones on mammary tumor initiation (studies evaluating established mammary tumors or mammary tumor cell lines were not included) and these studies were separated into mammary tumors induced by chemical carcinogens or transgenic expression of oncogenes based on the timing of soy administration. Regardless of when soy-based diets or purified isoflavones were administered, no consistent protective effects were observed in either carcinogen-induced or oncogene-induced mammary tumors. While some studies demonstrated that soy or purified isoflavones could reduce mammary tumor incidence, other studies showed either no effect or tumor promoting effects of soy products or isoflavones. Most importantly, only five studies found a decrease in mammary tumor incidence and six studies observed a decrease in tumor multiplicity, two relevant measures of the tumor preventative effects of soy or isoflavones. The variable outcomes of the studies examined were not completely surprising given that few studies employed the same experimental design. Future studies should be carefully designed to more accurately emulate soy consumption observed in Asian cultures including lifetime exposure to less refined soy products and potentially the incorporation of multigenerational feeding studies.

## 1. Introduction

It has been estimated that approximately 35% of human cancers are preventable through changes in lifestyle such as maintaining a healthy body weight, eliminating alcohol and tobacco, and adhering to cancer screening guidelines [1]. One lifestyle change that has been specifically associated with breast cancer is the consumption of high levels of dietary soy. Several epidemiologic studies found that women from cultures consuming high levels of dietary soy have an ~3-fold reduced risk of developing breast cancer compared to women that only consume small amounts of soy [2,3,4,5,6]. Several meta-analyses of the epidemiologic studies found that high consumption of soy reduced the risk of breast cancer in both pre- and post-menopausal women in studies performed on Asian populations but not on women from Western countries [6,7,8]. A 2019 meta-analysis found that individuals consuming high levels of isoflavones had similar breast cancer rates as those consuming low levels of isoflavones. When this meta-analysis only evaluated studies that reported the intake of soy foods, individuals consuming high levels of soy foods had a significant reduction in breast cancer risk compared to those in the low soy-food consumption group [9]. Therefore, although most studies suggest that consuming high levels of dietary soy may reduce the risk of developing breast cancer, there is no clear consensus.

However, some of this variation may stem from the population examined, types of soy products consumed (refined vs unrefined, fermented vs unfermented, and soy foods vs isoflavones), as well as the timing and duration of soy consumption. Ideally, randomized human prevention trials should be performed; however, these types of studies would take decades to complete, may raise ethical concerns of feeding developing fetuses or newborns specialized diets without their consent, and it would be difficult to maintain compliance to specific diets for such a long duration.

Since human randomized clinical trials are not feasible, animal models have been utilized with the most common being rodent models. Rodent mammary glands share features with the human breast including epithelial-lined ducts surrounded by myoepithelial cells, fibroblast and stroma [10]. In addition, both the mouse mammary gland and human breast respond to similar growth factors, cytokines and hormones during pubertal ductal development, alveologenesis and involution [10]. There are, however, differences in human and rodent mammary glands including the number of mammary glands, increased density of fibrous tissue in human breasts as well as more complex lobulo-alveolar structures in human breasts [11].

This review focuses on rodent models designed to assess the preventative actions of dietary soy products or purified isoflavones and includes papers published since 1995. For studies prior to 1995, please see the review by Barnes [12]. The efficacy of dietary soy or purified isoflavones in reducing breast cancer progression or tumor recurrence has been investigated but is beyond the scope of this review. While it is possible that soy isoflavones impact tumor cell progression or recurrence using similar mechanisms as those that prevent mammary tumor initiation, it is also possible that these tumor stages are regulated by distinct mechanisms and thus this review has focused on soy’s impact on tumor initiation and not progression or recurrence.

## 2. Soy and Isoflavones

Soy products are derived from soybeans and contain a number of compounds including protease inhibitors, phytosterols, saponins, and phytoestrogens known as isoflavones. Most of the work has focused on the isoflavone component of soybeans and the main isoflavones are genistein, daidzein and glycitein [13]. Isoflavones have chemical structures similar to mammalian estrogens and thus can bind to estrogen receptor-α (ERα) and ERβ [14,15,16]. The affinity of isoflavones for ERα and ERβ is also different with isoflavones preferentially binding to ERβ [17,18,19,20]. However, soy isoflavones have relatively weak estrogenic activity compared to endogenous estrogens [21,22] and thus, it is thought that dietary isoflavones partially impede endogenous estrogen signaling [14,23,24]. Since elevated lifetime estrogen exposure is a breast cancer risk factor [25], soy isoflavones may reduce breast cancer risk by suppressing the effects of endogenous estrogen. In addition to altering estrogen signaling, soy isoflavones have been reported to decrease lipid peroxidation and oxidative DNA damage through their antioxidant properties [24,26,27,28], promote apoptosis [26,29], inhibit angiogenesis [27,30,31,32] and regulate DNA methylation [33]. It should be noted that most mechanistic studies of soy isoflavones are performed in vitro using isoflavone concentrations exceeding 25 micromolar, while circulating levels of isoflavones in rodents and humans consuming soy-rich diets are typically less than 1–5 micromolar [29,31,32,34,35,36]. Moreover, in vitro studies cannot account for modifications of isoflavones during normal metabolism that can influence the relative abundance of isoflavone metabolites and their bioactivity [37]. While most of the studies have focused on the isoflavone components of soy, other soybean components such as protease inhibitors, phytosterols and saponins that may also influence breast cancer risk [38,39,40]. 

Not all sources of dietary soy are equal. Asian cultures typically consume minimally processed soybeans and more fermented soy products such as miso or tempeh where isoflavones are primarily in their aglycone form (genistein, daidzein and glycitein) [41]. In contrast, Western societies typically consume non-fermented soy products such as soy milk or tofu which contains isoflavones primarily in their glucosides form (genistin, daidzin and glycitin). In addition, soy products in North America often undergo extensive extraction and purification processes producing a product known as isolated soy protein (ISP) or soy protein isolate, which lacks most of the carbohydrates and fiber, leaving a product that is approximately 90% protein. This processing also influences the levels of isoflavones [42,43,44] and removes a number of soy compounds including protease inhibitors, phytosterols and saponins that may alter the protective properties associated with soybeans [42]. 

## 3. Rodent Mammary Tumor Models

One of the first and most common rodent models used to evaluate the impact of soy isoflavones on mammary tumor develop is the administration of 7,12-Dimethylbenzathracene (DMBA) to rats. DMBA is typically given as a single dose to pubertal female rats through injection or oral gavage and tumor incidence is close to 100% with tumor onset ranging from 8–21 weeks, depending on the concentration of DMBA administered [45]. Mammary tumors induced by DBMA are typically minimally invasive but remain hormone-dependent and thus are reasonable models for estrogen dependent breast cancers [46]. Other chemical carcinogens have also been used including *N*-methyl-*N*-nitrosourea (NMU) and ethyl methanesulfonate (EMS), which are typically provided as a single administration to pubertal female rats and 2-amino-1-methyl-6-penylimidazo[4–b]pyridine (PhIP) is administered orally to rats four times per week for two weeks. Although chemically induced rat mammary tumor models share features with human breast cancer they frequently induce mutations in *Hras* [47,48,49,50], a phenomenon not frequently observed in human breast cancers. However, a more recent study evaluating genetic alterations induced by DMBA in mice frequently observed mutations in *Pi3kca* and *Pten*, two relevant human breast cancer genes [51]. Chemical carcinogens are used less frequently in mice as mice are more resistant to chemically induced mammary tumors than rats and require multiple doses of chemical carcinogens [51].

With the generation of genetically modified mice, alterations in specific oncogenes or tumor suppressor genes became possible. The most widely used transgenic mammary tumor model is MMTV-*neu* transgenic mice. MMTV-*neu* mice express elevated levels of *EbbB2* (rodent equivalent of human *HER-2*) in mammary epithelial cells as well as other epithelial cells where the mouse mammary tumor virus (MMTV) promoter is expressed [52,53,54]. MMTV-*neu* mice develop mammary tumors with a median onset of 5–10 months [55,56,57] and these tumors have characteristics similar to human HER2+ tumors [58]. Another MMTV driven transgene that induces mammary tumor development is *Wnt1*. MMTV-*Wnt1* transgenic mice develop mammary tumors expressing both luminal and myoepithelial genes and cluster most closely with normal human breast tissue [58]. One limitation with MMTV-driven transgenes is that the oncogene is expressed at low levels throughout the animal’s lifespan. The MMTV promoter is responsive to steroid hormones [59,60,61,62] and thus its activity is highest during puberty and gestation with low levels of MMTV promoter activity prior to puberty. As it remains unclear when the initiating events for breast cancer occur, expression of an oncogene at all developmental stages may or may not accurately reflect oncogene expression in humans.

Two non-MMTV-driven transgenes have been used to study the impact of dietary soy on mammary tumor development: C(3)1/SV40 and Mt-*hGH*. C(3)1/SV40 transgenic mice express the simian virus 40 large T antigen driven by the rat prostatic steroid binding protein C3(1) 5′-flanking sequence. Female mice develop mammary tumors by 4 months of age and male mice develop prostate tumors by 1 year of age [63]. Mammary tumors from C(3)1/SV40 transgenic mice share characteristics with human basal-like tumors [58]. Mt-*hGH* transgenic mice express elevated levels of human growth hormone driven by a murine metallothionein promoter. These mice develop mammary tumors by 27–43 weeks of age [64]. Gene expression analysis has not been performed on these mammary tumors but the authors describe the tumors as malignant papillary adenocarcinomas [64].

In addition to the transgenic mice describe above, MTB-IGFIR transgenic mice [65] have also been used to investigate soy isoflavone’s impact on mammary tumorigenesis [66]. MTB-IGFIR transgenic mice overexpress the human insulin-like growth factor receptor (IGF-IR) in mammary epithelial cells in a doxycycline-inducible manner [65]. Mammary tumors rapidly develop in 100% of the mice and these mammary tumors cluster most closely with human basal-like breast cancers [67]. The MTB-IGFIR transgenic mice overcome one of the limitations of constitutive transgenic models (i.e., MMTV-driven transgenes) in that the IGF-IR transgene is only expressed when doxycycline is present and thus transgene expression can be initiated in pubertal or adult mice [65,68]. 

## 4. Timing of Soy Exposure

A key difference in the studies evaluating the impact of soy isoflavones on mammary tumor development in rodent models is the timing of soy isoflavone administration. Most of the early studies and even some of the more recent studies initiate the feeding of soy diets or purified isoflavones in postnatal rodents. This experimental approach would presumably emulate the human situation where children or adolescents switch their diet to one containing high levels of soy. However, Asian cultures, where reduced breast cancer rates are observed, would presumably consume high levels of soy at all stages of life including during pregnancy and lactation as well as during childhood, adolescence and adulthood. Therefore, lifetime exposure (gestation, lactation and postnatal development) may more accurately model the isoflavone consumption of cultures with reduced breast cancer rates.

Given the potential importance of the timing of soy exposure this review has been organized into two main sections: mammary tumor development following postnatal soy/isoflavone exposure, and mammary tumor development following lifetime soy/isoflavone exposure. There were also four studies that investigated soy exposure only during the perinatal developmental window, which have been included in this review. 

## 5. Mammary Tumor Development Following Postnatal Soy Isoflavone Administration

Twenty-three studies were identified that evaluated mammary tumorigenesis following the administration of soy-based diets or purified isoflavones during postnatal development; fourteen using chemical carcinogens in rats [69,70,71,72,73,74,75,76,77,78,79,80,81,82] and nine using transgenic mice [83,84,85,86,87,88,89,90,91]. For this review, postnatal administration was defined by the initiation of soy-based diets or isoflavones at weaning or later and the design of these studies and the main findings are summarized in Table 1. The impact of soy isoflavones on mammary tumor development were highly variable. This was not completely surprising given the differences in (1) the chemical carcinogen or oncogene used, (2) the source and concentration of soy products or purified isoflavones, and (3) the timing of soy/isoflavone administration. Within the 14 chemical carcinogen studies, 8 found that soy/isoflavones had some protective effect against mammary tumorigenesis (tumor incidence, latency, multiplicity or size) [70,71,72,73,74,76,79,82] but only 2 of these studies observed a truly protective effect against mammary tumor development as measured as a significant decrease in tumor incidence [71,72], while 3 studies demonstrated a significant reduction in tumor multiplicity [74,76,79]. Three of the studies using chemical carcinogens found that soy/isoflavones promoted mammary tumor incidence, multiplicity, or size [72,75,80]. 

The findings were also highly variable in the transgenic mouse models with four of the nine studies showing at least some protective effect against mammary tumorigenesis (tumor incidence, latency, multiplicity or size) [83,86,87,88], with two of these studies observing a significant decrease in tumor incidence [83,88]. Three of the studies found no effect [84,89,91] and three of the studies found that soy isoflavones promoted at least one mammary tumor property [83,85,90]. The transgenic data was more difficult to evaluate as often other characteristics (i.e., high fat diet, estrogen levels) and different concentrations of isoflavones were assessed in the same study. For example, the study by Zhang et al. [83] assessed the impact of soy on mammary tumor development in MMTV-*neu* mice with low (ovariectomized mice), normal or high (estradiol injection) levels of estrogen. In this study it was observed that diets high in soy increased tumor incidence in the low estrogen group, but the soy diet reduced tumor incidence in the high estrogen group (explaining why this study is referenced as soy-based diets having demonstrated both tumor protective and tumor promoting effects in the discussion above).

Genistein is the most studied individual soy isoflavone as it is the predominant isoflavone in soy and it can bind to estrogen receptors [92]. When evaluating only those studies that utilized purified genistein, eight studies [69,70,71,73,79,80,81,87,89] examined the impact of postnatal genistein administration on mammary tumor development with six of these studies performed on rats [69,70,71,73,79,80,81] and two in mice [87,89]. Three of the studies evaluating genistein in rats demonstrated some protective effect such as increased tumor latency or decreased tumor size [70,71,73]. Only one study demonstrated a significant decrease in tumor incidence [71]; however, a second study found a decrease in tumor multiplicity [74]. Of the two studies evaluating postnatal genistein administration in transgenic mice, one study observed an increase in tumor latency but no significant effect on tumor size, incidence or multiplicity [87], while the second study found no significant differences in tumor incidence or growth rate [89].

## 6. Mammary Tumor Development Following Lifetime Soy Isoflavone Administration

Fewer studies have evaluated the benefits of lifetime soy isoflavone exposure in rodent models of mammary tumor development and these studies are summarized in Table 2. Lifetime soy/isoflavone exposure was defined as soy/isoflavone administration during gestation, lactation and postnatal development. Only two studies using chemical induction of mammary tumors were found and both utilized NMU. While one study demonstrated a significant reduction in tumor incidence and prolonged tumor latency [93], the other study found no significant difference in tumor incidence [94].

There were also three studies using transgenic mice. One study using MMTV-*neu* mice found that mice fed a diet containing the isoflavone-enriched product, Prevastein, had reduced tumor incidence and prolonged tumor latency in the group that were fed a high-fat diet based on corn oil, but not in the group with a diet based on fish oil. Meanwhile, the other study found no impact on tumor incidence in MMTV-*neu* transgenic mice fed a high soy diet compared to controls [95]. In the study using MTB-IGFIR mice, tumor incidence was increased and tumor latency was decreased in MTB-IGFIR mice fed a diet containing 20% ISP compared to casein-fed MTB-IGFIR mice [66].

## 7. Mammary Tumor Development Following Perinatal Soy Isoflavone Administration

For this review, perinatal exposure was defined by soy or isoflavone administration between conception and weaning. Using this definition, there were six studies identified, four of which used chemical carcinogens in rats [94,97,98,99] and two studies that used MMTV-*neu* transgenic mice [91,96]. These studies are summarized in Table 3. None of the studies demonstrated a decrease in tumor incidence in soy/isoflavone treated rodents; however, three of the studies using chemical carcinogens found a decrease in tumor multiplicity [94,98,99]. One study using MMTV-*neu* transgenic mice found no significant difference in the tumor incidence or onset in Prevastein-treated mice compared to control mice [96] while the other study found that tumor multiplicity and size increased in the medium- and high-Prevastein groups compared to controls [91].

## 8. Conclusions and Future Considerations

The most appropriate measure of reduced breast cancer risk would be a reduction in tumor incidence (number of animals that develop mammary tumors) in rodents fed diets containing soy or isoflavones compared to rodents fed control diets. Of the 34 studies evaluated, only 5 demonstrated a significant reduction in tumor incidence in response to diets enriched with soy products or purified isoflavones. Tumor multiplicity was reduced in soy-fed mice in an additional 6 studies and thus 11 of 34 studies demonstrated that soy products or purified isoflavones reduced either the percentage of rodents that developed mammary tumors or the number of tumors that developed in each animal. When focusing on the studies that evaluated the administration of the purified soy isoflavone genistein, 4 of the 8 studies demonstrated that postnatal genistein reduced at least one tumor characteristic, with only 2 of these studies demonstrating a decrease in tumor incidence or multiplicity. Given that less than a third of the studies demonstrated a decrease in tumor incidence or multiplicity suggests that the current studies have failed to demonstrate a consistent, protective effect of soy isoflavones in preventing mammary tumor development.

Before concluding that either rodent models are unsuitable for breast cancer prevention studies or high levels of dietary soy do not reduce breast cancer risk, further research should be encouraged. However, a number of factors require careful consideration, including (i) the type of soy (unrefined/refined, fermented/unfermented, or purified isoflavones), (ii) the experimental model (mice, rats or another model such as primates), and (iii) when to initiate soy-based diets and how long these diets should be continued. Future studies should emulate the human data that most clearly implicates that the consumption of dietary soy reduces breast cancer risk, and these are the epidemiologic studies showing that lifetime/multigenerational exposure to diets containing unrefined, fermented soy products by some Asian cultures reduces breast cancer risk. Therefore, diets containing high levels of unrefined and possibly fermented soy products should be tested. The unrefined soy products would maintain most of the soybean components such as protease inhibitors, phytosterols and saponins that are typically lost during the refinement process. With respect to timing, lifetime exposure to soy-based diets should be the minimum and multigenerational exposure should be evaluated. No study investigating mammary tumor development following multigeneration soy exposure could be found, and it is possible that soy induces epigenetic alterations in the gametes of animals with lifetime soy exposure that then impacts the gene expression and tumor sensitivity of their offspring.

The most relevant animal model is more debatable. Although non-human primates likely represent the best model, large dietary studies in non-human primates genetically altered to express relevant human oncogenes or lacking key tumor suppressor genes are not feasible and would raise ethical concerns. Therefore, genetically altered mice or rats expressing inducible, tissue-specific oncogenes or inducible, tissue-specific knockouts utilizing known human tumor suppressor genes probably represent the most appropriate model since this system (i) uses known oncogenes and tumor suppressor genes and thus the mechanisms of tumor initiation will be more relevant to human breast cancer, (ii) permits oncogene expression or tumor suppressor gene ablation in postnatal animals which presumably emulates the timing of spontaneous activation of an oncogene or loss of a tumor suppressor gene in humans, and (iii) several inducible mouse models are currently available and their genetic similarity to different human breast cancer subtypes is often known.

However, a main concern with rodent models is that rodents and humans metabolize soy isoflavones differently [100,101]. These alterations in metabolism influence the amount of isoflavones present in their aglycone form and the amount of daidzein that is converted to equol [101]. Only approximately 30% of the western population produces equol [102] as a product of isoflavone metabolism while equol production has been reported in 50–60% of Asian adults [103,104,105,106]. One hundred percent of mice and rats produce equol [101]. Soy metabolism is further complicated by the fact that diets high in soy can alter the composition of the gut microbiome [107] and thus influence circulating isoflavone levels. Isoflavone levels in tissue has been poorly studied. Chang et al. (2000, genistein) evaluated the levels of genistein in rats and found genistein in a number of tissue including the mammary gland and tissue typically contain a higher percentage of the aglycone form of genistein than the plasma [98,108]. Only a small number of studies have evaluated isoflavone levels in human breast tissue, and the limited data suggests that isoflavones or particular metabolites do not preferentially accumulate in breast tissue [41]. Given the differences in isoflavone metabolism, future animal studies should measure plasma and tissue isoflavone levels so isoflavone levels and composition in animal models can be compared to those achievable in humans.

## Figures and Tables

**Table 1:** Postnatal Soy/Isoflavone Administration.

Species	Isoflavone Diet/Timing	Tumor Inducer	Main Finding	Refs
rat	0.25 g/kg or 1 g/kg of daidzein or genistein separately or 1 g/kg of both daidzein and genistein, PND35-EOS	1 oral dose, 80 mg/kg body weight DMBA at PND50	No significant difference in tumor incidence or size compared to control diet	[69]
rat	500 ppm genistein in diet, PND15–30, PND15–30 and PND55-EOS or PND55-EOS	1 oral dose, 10 mg DMBA at PND48	Tumor onset delayed only in group fed genistein PND15–30 and PND55-EOS compared to control diet. No significant difference in tumor incidence	[70]
rat	2 mg/kg body weight, genistein orally, PND42-EOS	1 oral dose, 80 mg/kg body weight DMBA at PND55	Tumor incidence and size significantly reduced in genistein group compared to controls	[71]
rat	3.24 mg total isoflavones/g protein in diet of lean or obese rats, PND42-EOS	1 oral dose, 65 mg/kg body weight DMBA at PND50	Tumor incidence significantly reduced in lean soy fed rats vs lean casein fed rats yet tumor incidence significantly higher in obese soy-fed rats vs obese casein fed rats. No significant differences in tumor onset or multiplicity	[72]
rat	Genistein 20 mg/kg body weight, daidzein 20 mg/kg body weight or genistein + daidzein 20 mg/kg each), oral 1 week before DMBA-EOS	1 injection, 25 mg DMBA, exact age not defined	Genistein alone, daidzein alone and the combination significantly reduced tumor size compared to control mice. Tumor incidence appeared to be reduced, especially in combination group but no significance was indicated.	[73]
rat	Isoflavone-deprived soy peptide, PND28-PND56 and PND63-EOS	1 oral dose, 50 mg/kg body weight DMBA at PND56	Tumor latency was significantly increased, and tumor size and multiplicity were significantly decreased in isoflavone-deprived soy group vs control group	[74]
rat	Soy milk PND50-EOS	1 oral dose, 5 mg DMBA at PND49	Tumor incidence significantly higher in soy milk group compared to water group; no significant differences in tumor multiplicity or size	[75]
rat	20% soy protein, PND25-EOS	1 oral dose, 80 mg/kg body weight DMBA at PND50	Tumor onset significantly delayed, and tumor multiplicity significantly reduced in soy group vs control group but no difference in tumor incidence at study endpoint	[76]
rat	Soy-free diet with 0.35% or 0.7% (w/w) SOYSELECT (12% isoflavones and 35% saponins), PND21-EOS	1 oral dose, 80 mg/kg body weight DMBA at PND50	No significant differences observed at study endpoint	[77]
rat	0.03, 0.4 or 0.81 mg/g diet isoflavones, PND36-EOS	1 oral dose, 10 mg DMBA at PND50	No significant differences in tumor incidence, onset, multiplicity or burden	[78]
rat	200 mg/kg diet genistein, 200 mg/kg diet daidzein, 100 mg/kg diet each of genistein + daidzein, 160 g/kg diet SPI or 160 g/kg diet SPI depleted of isoflavones, PND43-EOS	1 oral dose, 15 mg DMBA at PND50	Tumor multiplicity significantly reduced in daidzein and both SPI diets; no significant difference in tumor incidence, mean latency or size in any of the diets	[79]
rat	1 mg/kg body weight genistein injected daily, PND45-EOS	1 injection, 40 mg/kg body weight NMU at PND45	Tumor multiplicity and size significantly elevated in genistein group vs control group	[80]
rat	0.03 or 1 mg/g of genistein in soy free diet or soy containing basal diet PND28-EOS	Oral, 10^−3^ M EMS in drinking water, PND28-PND112	no significance difference in tumor incidence, size or latency compared to control group	[81]
rat	100 g soymilk powder/kg diet alone or with 2 g/kg diet *Lactobacillus casei* in a high fat diet, PND35-EOS.	oral, 85 mg/kg PhIP, 4 x/week for 2 weeks, PND42–56	according to Table 2, no significant differences in tumor incidence, multiplicity or size in soymilk vs control, however Figure 1 indicates that tumor multiplicity significantly reduced at study endpoint. The combination of soymilk and *Lactobacillus casei* significantly reduced tumor multiplicity	[82]
mouse	Soybean diet (40% soybean meal), PND49-EOS	MMTV-*neu*, low estrogen (ovariectomy), normal estrogen (untreated), and high estrogen (estradiol injection)	Tumor incidence significantly increased in soy-fed, low estrogen group but tumor incidence significantly reduced in soy-fed, high estrogen group. No significant differences in tumor latency or size	[83]
mouse	21.7% soy protein isolate, PND60-EOS	MMTV-*neu* (did not consider *ERΔ3*/*neu* mice)	No significant effect on tumor incidence or latency in soy-fed MMTV-*neu* mice compared to MMTV-*neu* mice fed a control diet	[84]
mouse	0.004%, 0.02% or 0.06% wt/wt Prevastein (46.19% wt/wt isoflavones), PND25-EOS	MMTV-*neu* fed a Western diet (high fat, moderate fiber, low calcium)	Significant increase in tumor multiplicity and size in highest isoflavone group compared to control group; no differences in medium or low isoflavone group and no significant differences in tumor incidence between any of the groups	[85]
mouse	Purina 5001 (soy diet), PND28-EOS	MMTV-*neu* implanted with 0.5 mg, 60-day constant release estrogen pellet	Tumor onset significantly delayed in soy group for both placebo and estrogen pellet mice vs control mice; no significance different in tumor incidence was reported	[86]
mouse	250 mg/kg genistein, 250 mg/kg daidzein, NovaSoy, PND56-EOS	MMTV-*neu*, 1 pregnancy and 2 weeks of lactation	Tumor latency delayed in all isoflavone groups, tumor growth, incidence, multiplicity and size not affected	[87]
mouse	Supro 670 with low or high isoflavone (0.2 and 1.81 mg isoflavone/g protein isolate), PND28-EOS	MMTV-*neu* on high fat diet	No significant difference in tumor incidence, onset, multiplicity or size	[91]
mouse	430 mg isoflavones/kg diet, PND21-EOS	MMTV-*Wnt1*	Tumor incidence and latency reduced in isoflavone group	[88]
mouse	250 mg/kg genistein, PND28-EOS	C(3)1-SV40	No effect on tumor incidence or growth rate	[89]
mouse	32 mg/kg or 972 mg/kg isoflavones, PND22-EOS	MT-*hGH*	Tumor latency reduced, and tumor size increased in high isoflavone group	[90]

PND = post-natal day; EOS = end of study; DMBA = 7,12-Dimethylbenzathracene; NMU = *N*-methyl-*N*-nitrosourea; PhIP = 2-amino-1-methyl-6-phenylimidazo[4,5-*b*]pyridine.

**Table 2:** Lifetime Soy/Isoflavone Administration.

Species	Isoflavone Diet/Timing	Tumor Inducer	Main Finding	Refs
rat	ISP, gestation day 4-EOS^1^	1 injection of 50 mg/kg body weight NMU at PND50	Tumor incidence reduced, and latency increased in SPI group; tumor multiplicity not affected	[93]
rats	ISP, gestation day 4-EOS	1 injection of 50 mg/kg body weight NMU at PND51	No significant differences in tumor incidence or multiplicity	[94]
mice	90 mg/kg Prevastein (46.19% wt/wt isoflavones) 2 weeks prior to mating-EOS	MMTV-*neu* on high fat diet with either corn oil or fish oil	Decreased tumor incidence and increased tumor latency in isoflavone group with corn oil but no significant differences in group with fish oil	[96]
mice	Soy containing 4RF21, breeding-weaning and then 4RF21, SPI or isoflavone poor concentrate, PND21-EOS	MMTV-*neu*	No difference in tumor incidence	[95]
mice	20% ISP, breeding-EOS	MTB-IGFIR (IGF-IR induced at PND45 or PND100)	Tumor onset reduced, and incidence increased in ISP group	[66]

^1^ EOS = end of study; PND = post-natal days; ISP = isolated soy protein; NMU = *N*-methyl-*N*-nitrosourea.

**Table 3:** Perinatal Soy/Isoflavone Administration.

Species	Isoflavone Diet/Timing	Tumor Inducer	Main Finding	Refs
rat	250 mg daidzein/kg diet 2 week prior to mating-weaning	1 oral dose, 40 mg DMBA at PND50	No significant differences in tumor onset or incidence	[97]
rat	25 or 250 mg genistein/kg diet conception-weaning	1 oral dose, 80 mg/kg DMBA at PND50	Tumor multiplicity reduced in isoflavone group	[98]
rat	20 ug genistein injected on PND7, 10, 14, 17 and 20	1 injection, 10 mg DMBA at PND45	No significant effect on tumor latency or incidence but multiplicity and growth rate significantly lower in genistein group vs control group	[99]
rats	SPI, gestation day 4-EOS	1 injection of 50 mg/kg body weight NMU at PND51	No significant differences in tumor incidence but multiplicity significantly reduced	[94]
mice	0, 18, 90 or 270 mg/kg Prevastein (46.19% wt/wt isoflavones), conception-weaning	MMTV-neu on normal or high fat diet	No significant difference in tumor incidence but tumor multiplicity and size significantly increased in medium and high isoflavone group	[91]
mice	90 mg/kg Prevastein (46.19% wt/wt isoflavones) 2 weeks prior to mating-weaning	MMTV-*neu* on high fat diet with either corn oil of fish oil	No differences in tumor incidence or onset	[96]
